# The NICOTINURSE Trial: Protocol for a stepped-wedge trial to implement nurse-led smoking cessation support in a tertiary care hospital

**DOI:** 10.18332/tid/231136

**Published:** 2026-10-31

**Authors:** Anusha Savundrarajan, Anne-Isabelle Tropeano, Aurelie Vilfaillot, Marie-Laure Delaporte, Anne-Laurence Le Faou

**Affiliations:** 1 Centre ambulatoire d'addictologie, Hôpital Européen Georges Pompidou, Paris, France; 2 Clinical Research Unit, Georges Pompidou European Hospital, AP-HP Centre – Paris Cité University, Paris, France; 3 Vascular Medicine/Hypertension Department, Georges Pompidou European Hospital, AP-HP Centre-Paris Cité University, Paris, France; 4 Centre Ambulatoire d’Addictologie, AP-HP Centre - Université Paris Cité, Paris, France

**Keywords:** smoking cessation, nicotine addiction, nicotine replacement therapy, hospital care, nurses training

## Abstract

Smoking cessation support is still lacking during hospital stay, leading smokers to suffer from withdrawal symptoms and missing opportunities to help them quit. In this context, NICOTINURSE is a stepped-wedge cluster randomized trial aimed to evaluate the effectiveness of a nurse-led support and nicotine replacement therapy (NRT) prescription during smokers’ hospital stay in four departments of a university hospital on one-month abstinence after discharge with a follow-up of quit attempts at 3 and 6 months. This is a stepped-wedge cluster trial, including four hospital departments. Inpatients who smoke in the experimental group will receive structured counseling and NRT prescribed by trained nurses in smoking cessation before discharge, while smokers in the control group receive standard care. The primary outcome is one-month abstinence post-discharge confirmed biochemically. Secondary outcomes, assessed at 1, 3, and 6 months post-discharge, include: abstinence, mean number of cigarettes smoked daily, quality of life scale, anxiety and depression symptoms score, addiction center use, and prescribed NRT during hospitalization and at discharge. Nurse job satisfaction will be measured at the beginning and at the end of the study. Statistical analyses will compare intervention and control groups using models appropriate for the stepped-wedge cluster randomized design (two-sided α = 0.05). Based on an expected 1-month abstinence rate of 15% in the control group and 30% in the intervention group, with 80% power, an intraclass correlation coefficient of 0.30, and 5% loss to follow-up, a total sample size of 442 participants is required. All data collected about the study participants will be anonymized. Results will be communicated to participants, hospital departments’ staff and other relevant groups without any restrictions.

**CLINICAL TRIAL REGISTRATION:**

The study is registered on the official website of ClinicalTrials.gov

**IDENTIFIER:**

NCT06789536

## INTRODUCTION

Tobacco use remains the leading preventable cause of death in France and is responsible for approximately 13% of avoidable deaths each year^1^. Yet, smoking reduces life expectancy by an average of 10 years^2^, and despite this significant impact on public health, 23% of people aged 15–75 years smoked daily in 2023 in France^3^. Nevertheless, behavioral interventions as well as medications such as nicotine replacement therapy (NRT), varenicline, and bupropion have proven effective to quit^4^, with NRT and varenicline being covered by the health insurance system. Since 2016, registered nurses in France have been legally authorized to prescribe NRT, without requiring certification or additional training in smoking cessation, but this possibility is rarely used in practice^5^.

The benefits of involving nurses in smoking cessation support are well documented. A Cochrane review showed that nurse-led interventions increased quit rates by 30% across clinical settings^6^. Moreover, intensive in-hospital cessation support with NRT prescription offers significant benefits^7^. Finally, nurses might play a key role in smoking cessation in specialized hospital settings by managing NRT dosing and follow-up care^8^.

Implementing nurse-led smoking cessation care in hospital settings remains challenging because smoking assessment and cessation support are often not integrated into routine care, owing to competing clinical priorities, limited time, and variable staff training. To address these challenges, a survey was conducted in 2016 at the Georges Pompidou European Hospital (HEGP), a university hospital in Paris, showing that smoking status was recorded in only 30% of patients’ electronic medical records (EMRs)^9^. Consequently, since 2021, smoking status has been systematically recorded in EMR by nursing staff at admission^10^. Around 12% of inpatients were smokers in 2023, with 83 individuals present daily among the hospital’s 691 beds. The HEGP addiction center team members, with their *Equipe de liaison et de soins en addictologie* (ELSA)^11^, provide bedside counseling to inpatients and recommendations to the medical teams when being contacted by the departments’ staff. Given the smoking prevalence and the short duration of hospital stays, the HEGP addiction center team cannot systematically provide care to every smoker hospitalized in HEGP departments. However, although nurse-led smoking cessation interventions have shown potential benefits, evidence remains limited regarding how to effectively integrate such interventions into routine hospital care and ensure their implementation across different clinical settings. To address this limit, involving nursing teams in daily smoking cessation support within specialized hospital departments appears both necessary for hospitalized smokers at risk of withdrawal symptoms and promising for improving cessation care. This requires empowering nurses with the skills and tools needed to deliver structured smoking cessation support.

Therefore, our research aims to implement a nurse-led smoking cessation intervention by training nurses in smoking cessation care in HEGP hospital, including NRT prescriptions and post-discharge follow-up, to establish sustainable cessation practices within nursing teams. This implementation trial will evaluate both smoking cessation outcomes and implementation outcomes, including nurses’ satisfaction with the intervention, the uptake of NRT prescriptions, and the integration of this approach into routine clinical practice over time.

### Research hypothesis

We hypothesize that training the nursing team to provide smoking cessation support and NRT prescriptions might increase quit attempts and one-month abstinence rates after discharge.

## METHODS

NICOTINURSE is designed as a stepped-wedge cluster randomized trial. The protocol was developed in accordance with the CONSORT statement, including its extension for stepped-wedge cluster randomized trials^12,13^. The protocol development was also guided by the recommendations of the SPIRIT checklist^14^.

### Study description

NICOTINURSE trial will be conducted across four departments: intensive care cardiology unit (ICCU)/cardiology department, nephrology department, pulmonology department, and arterial hypertension/vascular medicine (AH/VM) department. These departments were selected because smoking is a risk factor among their patients’ populations, making nurse-led intervention particularly relevant.

### Stepped-wedge cluster randomized trial design

NICOTINURSE is an open, cluster-randomized, stepped-wedge trial. In this design, departments represent 4 clusters, corresponding to nurse teams of each department. At the start of the study, no team will benefit from training. Every 5 months, a team will be trained, with the training taking place over a 2-week period. Once a team is trained, the department remains in the experimental group until the study ends. The order in which departments undergo training will be randomized. This design consists of five time periods, including a baseline period corresponding to usual care in the 4 departments (Figure 1).

**Figure 1:**
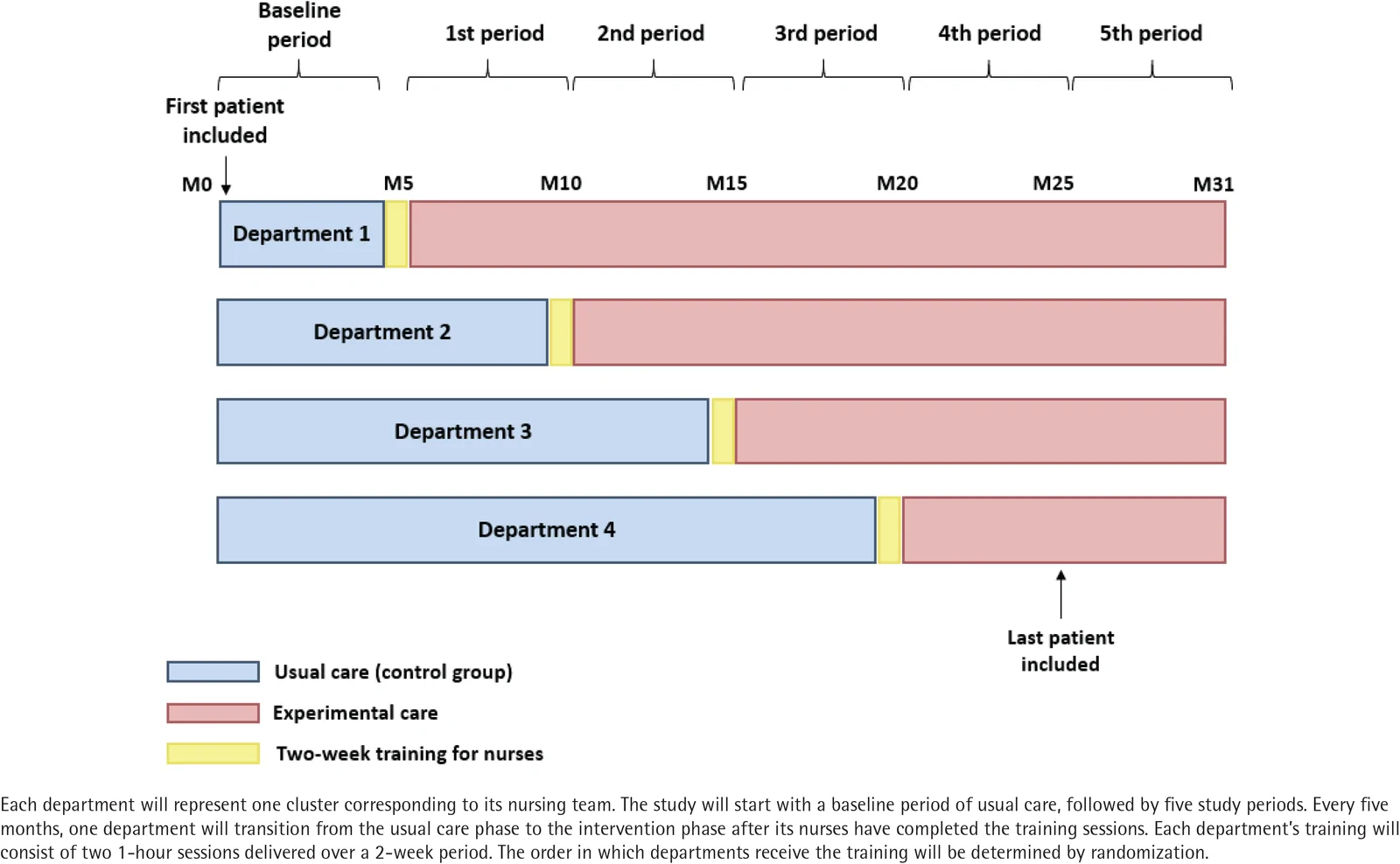
Stepped-wedge cluster randomized design of the NICOTINURSE trial, September 2025–April 2028. The study will be conducted at Georges Pompidou European Hospital (HEGP), Paris, France, across 4 departments: the Intensive Care Unit (ICCU)/Cardiology department, Nephrology department, Pulmonology department, and Arterial Hypertension/Vascular Medicine (AH/VM) department

The stepped-wedge design was chosen to minimize contamination bias between trained and untrained nurses within the same department, as all departments eventually receive the intervention.

The order in which departments undergo training will be determined using a randomization sequence generated with SAS software^15^ by a statistician blinded to the identity of the departments. The randomization sequence will be securely stored by the study sponsor (AP-HP). Owing to the nature of the intervention, blinding of nurses, participants, and outcome assessors is not feasible. However, outcome assessors (the scientific research coordinator [ALLF], the research assistant [AS], and the replacement research assistant) can remain blinded from the first study period onwards, as outcomes and secondary endpoints are based on participant-reported measures and objective measurements (expired CO), rather than on the smoking-cessation care delivered or the department in which participants were hospitalized.

### Usual care

The usual care, when provided, is either coordinated by ELSA, reachable via a dedicated phone number, or provided by medical doctors of each specialized department. The ELSA members document the EMR on smoking behaviors^16^, prescribe NRT during hospital stay as well as upon discharge, and arrange post-discharge referrals (addiction centers, ambulatory health professionals or national quit line)^17^.

### Description of intervention

During the NICOTINURSE study, nurses from the 4 departments will be trained in smoking cessation care, according to the study’s five periods (Figure 2). The training will be delivered by the scientific research coordinator (ALLF), a member of the HEGP addiction center team.

**Figure 2:**
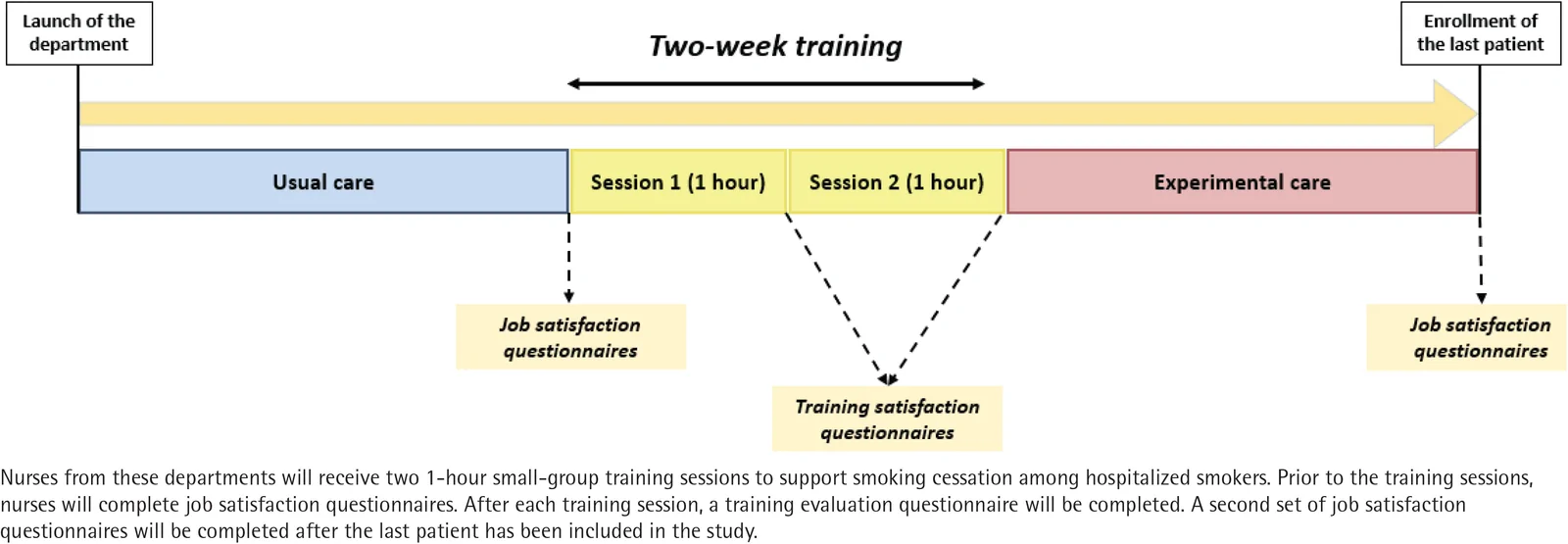
Schedule of the two-week nurse training period in the NICOTINURSE stepped-wedge cluster randomized trial

The 4 nursing teams will undergo two one-hour training sessions, delivered in small groups, to facilitate interaction.

Nurses will be equipped with the skills necessary to communicate with the smoker, such as motivational tools and a non-judgmental way of questioning smokers^18^, informing on NRT use and adapted doses^19^, as well as arranging follow-up^17^. Table 1 details the training sessions’ contents. Successful completion of the training will be assessed by attendance at both sessions and completion of a training evaluation questionnaire. Following completion of the training, access to NRT prescription through the EMR will be authorized by the IT team. During the initial implementation phase, the addiction center team will support nurses with the first NRT prescriptions. Prescription data and smoking cessation support delivered by nurses will be monitored through the EMR.

**Table 1:** Nurse training workshops* that will be delivered as part of the NICOTINURSE stepped-wedge cluster randomized trial to support smoking cessation in hospitalized smokers. Training will be implemented sequentially according to the randomized stepped-wedge schedule during the first two weeks of February 2026, July 2026, December 2026, and May 2027

*Session*	*Training program outline*	*Instructors*	*Deliverables*
1	How to deal with smokers in practice? Cigarette composition: Key components of tobacco smoke (carbon monoxide, irritants, carcinogens) and the effects of inhaled nicotine. Types of dependence: Physical: mechanisms, withdrawal, cravings, past quit attempts, Heaviness of Smoking Index (HSI). Behavioral: habits, environments. Psychological: stress, solitude, stimulation. CO measure at patient admission: motivational and educational purpose. Smoking cessation support (French National Authority for Health, 2015): Communication with smokers. NRT available in hospital: forms (nicotine patches and oral forms), use, effectiveness, potential side effects. Presentation of the validated pharmaceutical protocol. Adaptation of prescribed nicotine doses Two quizzes	Addiction Center: the scientific research coordinator (ALLF) and the research assistant (AS)	Minnesota Job satisfaction questionnaire^23^ Training validation form
2	HEGP tobacco file: key data to collect for tailored cessation support (e.g. previous quit attempts, tobacco dependence measured by the HSI, e-cigarette use with/without nicotine, nicotine liquid dosage, daily nicotine liquid quantity), other nicotine products, co-addictions. EMR: NRT prescription during hospital stay and at discharge. Discharge process: NRT prescription, a discharge summary to the patient’s primary care providers. Brochure ‘Tobacco, not for me!’ written by the Addiction Center team members. Clinical practice: Relevant data on smokers suffering from diseases hospitalized in each specialized department, case discussion with quizzes, prescription modalities during hospital stay and at discharge. Two quizzes	Addiction Center (ALLF, AS)	Training documents including additional videos for night-shift staff Training validation form

* The workshops will take place in four departments at Georges Pompidou European Hospital (Paris, France): the Intensive Care Cardiology Unit (ICCU)/Cardiology, Nephrology, Pulmonology, and Arterial Hypertension/Vascular Medicine (AH/VM) departments. Approximately 50 registered nurses will attend two 1-hour small-group training sessions delivered over a 2-week period by the scientific research coordinator (ALLF). CO: carbon monoxide; NRT: nicotine replacement therapy. HEGP: Georges Pompidou European Hospital. HIS: heaviness of smoking index. EMR: electronic medical records.

### Participants

*Eligibility criteria*

The study population consists of current smokers, defined as people with active tobacco use^20^, hospitalized in the participating HEGP departments. Eligible participants will be identified through systematic smoking status assessment at admission by nurses. In cases where smoking status has not been recorded, the research assistant will screen the list of patients recently admitted to identify potential smokers and will inform the nurses, who will approach eligible patients and propose study participation.

Eligible participants are adult patients hospitalized in one of the participating departments, identified as current smokers, residing in the Île-de-France region, and able to provide informed consent. Patients not affiliated with the French national health insurance system, under legal protection, pregnant or breastfeeding women, or those meeting other predefined exclusion criteria are not eligible. Detailed inclusion and exclusion criteria are described in Table 2.

**Table 2:** Inclusion and exclusion criteria for hospitalized adult smokers eligible for participation in the NICOTINURSE stepped-wedge cluster randomized trial

*Inclusion criteria*	*Exclusion criteria*
Age ≥18 years	Not affiliated with the French national health insurance system
Active tobacco use	Covered by State Medical Aid
Hospitalized in one of the following HEGP departments during the study period: ICCU/cardiology, nephrology, pulmonology, and arterial hypertension/vascular medicine	Covered by legal protection measures
Resident of the Île-de-France region (Paris region)	Women aged 18–49 years who have tested positive for pregnancy or are breastfeeding
Proficient in French	Refused to participate in the study
Written informed consent provided	

### Primary outcome

The primary outcome is defined as one-month abstinence after hospital discharge that captures the high risk of early relapse and is a strong predictor of long-term abstinence, increasing fivefold after the first four weeks of abstinence^21^. Abstinence is defined as no tobacco use in the 7 days preceding the follow-up visit. Smoking status will be biochemically verified in all participants at 1, 3, and 6 months after hospital discharge using exhaled carbon monoxide (CO), with abstinence confirmed by a CO level of ≤10 parts per million (ppm)^22^.

### Secondary outcomes

Secondary outcomes include both smoking-related and psychosocial measures to evaluate the impact of the intervention at 1, 3, and 6 months after discharge (Table 3). The nurses’ job satisfaction score^23^ is completed before training and at the study end (Table 1).

**Table 3:** Study outcomes assessed at 1, 3, and 6 months after hospital discharge in the NICOTINURSE stepped-wedge cluster randomized trial evaluating nurse-led smoking cessation support among hospitalized smokers, September 2025 – April 2028

*Measure*	*Methods of data collection*	*Source*	*Time point*
**Primary outcome of intervention**			
Smoking abstinence	Structured interview carried out at the hospital at one-month follow-up, and confirmed by CO ≤10 ppm	Smokers hospitalized at HEGP in one of the four study departments	One month after hospital discharge
**Secondary outcomes of intervention**			
Average number of cigarettes smoked per day	Structured interviews carried out at the hospital or over the phone.	Smokers hospitalized at HEGP in one of the four study departments	1, 3, and 6 months after hospital discharge
Proportion of patients who report a reduction of at least 50% in their daily cigarette consumption	Structured interviews carried out at the hospital or over the phone.	Smokers who were hospitalized at HEGP in one of the four study departments	1, 3, and 6 months after hospital discharge
Proportion of patients achieving complete smoking abstinence	Self-declared and confirmed by CO ≤ 10 ppm.	Smokers who were hospitalized at HEGP in one of the four study departments	3 and 6 months after hospital discharge
Quality of life assessed using the SF-12 questionnaire	Structured interviews carried out at the hospital or over the phone.	Smokers who were hospitalized at HEGP in one of the four study departments	1, 3, and 6 months after hospital discharge
Anxiety and depression scores assessed using the Hospital Anxiety and Depression score (HAD)	Structured interviews carried out at the hospital or over the phone.	Smokers who were hospitalized at HEGP in one of the four study departments	1, 3, and 6 months after hospital discharge
Proportion of patients reporting having visited an addiction center	Structured interviews carried out at the hospital or over the phone.	Smokers who were hospitalized at HEGP in one of the four study departments	6 month post-discharge
Proportion of patients receiving a nurse-initiated NRT prescription	Collected in the EMR	Hospital’s electronic prescribing system	During hospitalization and at discharge

Participants will be recruited from four departments at Georges Pompidou European Hospital (Paris, France): the Intensive Care Cardiology Unit (ICCU)/Cardiology, Nephrology, Pulmonology, and Arterial Hypertension/Vascular Medicine (AH/VM) departments. All data collection procedures, including questionnaire administration and CO measurement during study visits, performed by the research assistant. CO: carbon monoxide; NRT: nicotine replacement therapy. HEGP: Georges Pompidou European Hospital. HIS: heaviness of smoking index. EMR: electronic medical records. SF-12 questionnaire: Quality of life questionnaire.

### Harms

Adverse events potentially related to NRT will be systematically recorded throughout the study. Expected adverse events include skin reactions at the patch application site, insomnia, headache, gastrointestinal disorders (including abdominal pain, nausea, vomiting, and bowel disturbances), dry mouth, muscle pain, and any other reported symptoms. Each adverse event will be documented using a dedicated adverse event form and managed according to standard clinical practice. Given the well-established safety profile of NRT, no specific serious adverse events related to the intervention are anticipated. However, any adverse event reported during the study will be recorded.

### Participant timeline

At the time of inclusion (initial visit, V0), the study will be presented to the patient by an investigator, nurse or medical doctor, and the patient will be given time to provide written informed consent. For women of childbearing age, a pregnancy test will be performed to confirm eligibility. On the day of inclusion, demographic characteristics, medical history, and smoking behaviors will be collected. Self-administered questionnaires, the SF12 quality of life^24^ and the Hospital Anxiety and Depression scale (HAD), will also be provided to the smoker^25^, with the exhaled CO measure being performed. In the control group, patients will receive usual care. In the experimental group, smokers will receive counseling from trained nurses on NRT forms in order to determine the most suitable option to be prescribed during hospital stay and at discharge. NRT will be prescribed through the hospital’s electronic prescribing system and recorded in the patient’s EMR. The prescribed NRT will be retrieved from the automated medication dispensing cabinet available in each department and provided to the patient during the hospital stay.

At discharge (V1), patients will receive an NRT prescription for a one-month period with a dosage adjustment guide and a dedicated brochure ‘Tobacco, not for me!’.

Patients will return for follow-up visits at 1 (V2), 3 (V3), and 6 months (V4). To minimize loss to follow-up, participants will receive reminders before each scheduled visit, and follow-up appointments will be adapted according to patients’ availability. If a visit cannot be performed in person, patients will be contacted by phone to complete the follow-up assessment remotely. Table 4 outlines the content of these visits.

**Table 4:** Participant timeline of visits (V0–V4) for hospitalized smokers included in the NICOTINURSE stepped-wedge cluster randomized trial, September 2025 – April 2028

	*V0 Inclusion*	*V1 Hospital discharge*	*V2 1 month ± 3 days after hospital discharge*	*V3 3 months ± 4 days after hospital discharge*	*V4 6 months ± 6 days after hospital discharge*
Patient information and consent collection	✓				
Assessment of inclusion and exclusion criteria	✓				
Demographic data and medical history	✓				
HEGP Tobacco treatment record	✓				
Quality of life questionnaire (SF12)	✓		✓	✓	✓
HAD questionnaire	✓		✓	✓	✓
Tobacco use questionnaire	✓		✓	✓	✓
Exhaled CO measurement	✓		✓	✓	✓
Intervention according to the nurse’s group		✓			

A compensation payment of €30 will be paid to the patient for follow-up consultations. Participants will be recruited from four departments at Georges Pompidou European Hospital (Paris, France): the Intensive Care Cardiology Unit (ICCU)/Cardiology, Nephrology, Pulmonology, and Arterial Hypertension/Vascular Medicine (AH/VM) departments. HEGP: Georges Pompidou European Hospital. HAD: Hospital Anxiety and Depression score. CO: carbon monoxide.

### Sample size

Based on prior studies on smoking cessation in hospitalized patients^26^, the expected one-month abstinence rate would reach 15% in the control group and 30% in the intervention group. Based on a Wald Z-test, and assuming a two-sided alpha of 0.05, 80% power, and an intraclass correlation coefficient^27^ of 0.3, the estimated required sample size is 420 patients. Accounting for a 5% loss to follow-up, the final target sample size is 442 patients.

Each cluster will enroll about 111 patients over the study. With time periods set at 5 months each, the recruitment phase would span 25 months. Including a 6-month follow-up per patient and additional time for data analysis and reporting, the study duration is estimated at 36 months.

### Feasibility of the study

At least ten smokers are expected to be admitted weekly in the 4 departments, based on a preliminary review of smoking status data recorded in the EMR of patients admitted to these departments. Assuming about half to provide consent to participate, the recruitment capacity should be adequate to achieve the target sample size within the planned timeline.

### Data collection and analyses

Data from smokers who provide informed consent will be documented in an electronic case report form (eCRF) via CLEANWEB, while data concerning nurses will be recorded in another eCRF. Data entry will be performed by the research assistant. Data will be coded using unique participant identification numbers. Access to the database will be restricted to authorized study personnel, and data will be stored securely according to institutional and regulatory requirements. Data quality will be ensured through regular monitoring procedures, including verification of data completeness and consistency, and review of missing or inconsistent data.

All analyses will be performed according to the intention-to-treat principle, with participants analyzed according to the intervention period of their department at the time of inclusion. Missing data will be described, and analyses will be performed using available data. If the proportion of missing data is substantial, appropriate sensitivity analyses will be considered. Statistical analyses will be performed using SAS version 9.4 (SAS Institute, Cary, NC, USA) or R software (R Foundation for Statistical Computing, Vienna, Austria)^15^. A two-sided significance level of 0.05 will be used.

### Ethics and confidentiality

*Ethics approval*

The research protocol has been approved by the French ethics committee *CPP Nord Ouest I* in 2024 (Approval number: 24.03798.000318) and was registered at ClinicalTrials.gov (NCT06789536). Any substantial protocol amendments will be submitted to the ethics committee for review and approval before implementation. Approved amendments will be communicated to all study personnel and documented in the study records.

*Confidentiality*

All study data will be pseudonymized to protect participants’ confidentiality.

### Dissemination plan

Results will be disseminated through presentations at scientific conferences and publications in peer-reviewed journals.

## DISCUSSION

The benefits of smoking cessation are established, in terms of improving quality of life, disease evolution, and psychological indicators. For patients, additional benefits might include greater comfort when attempting to quit, associated with NRT use during hospital stay and after discharge^28^, as well as enhanced support to enable them to stop smoking and avoid early relapse.

For nurses, the training would allow them to gain new skills, prescribe autonomously, and enhance their decision-making latitude. The potential impact of the intervention on nurses’ work experience will be assessed using a job satisfaction questionnaire administered before the training and after inclusion of the last patient. To facilitate implementation despite competing clinical priorities, nurses will be supported by the research assistant when needed. When nurses are unable to prescribe NRT, they will seek support from ELSA.

## CONCLUSIONS

At hospital level, this intervention could contribute to the implementation of nurse-led NRT prescription in other departments, improving access to cessation support and enhancing health outcomes and quality of life for inpatients. Integrating this model into routine nursing practice would also ensure better continuity of care post-discharge.

## Data Availability

Data sharing is not applicable to this article as no new data were created.
